# Picosecond Laser Etching of Glass Spiral Microfluidic Channel for Microparticles Dispersion and Sorting

**DOI:** 10.3390/mi16010066

**Published:** 2025-01-07

**Authors:** Rong Chen, Shanshan He, Xiansong He, Jin Xie, Xicong Zhu

**Affiliations:** 1 College of Mechanical and Electrical Engineering, Guangdong University of Science and Technology, Dongguan 523668, China; rongchen338@163.com; 2 School of Mechanical and Automotive Engineering, South China University of Technology, Guangzhou 510640, China; xiangyangsiji37@gmail.com (S.H.); hxs712428@foxmail.com (X.H.); xczhu0523@163.com (X.Z.)

**Keywords:** glass microfluidic channel, microfluidic chip, laser etching, particle sorting

## Abstract

In microfluidic chips, glass free-form microchannels have obvious advantages in thermochemical stability and biocompatibility compared to polymer-based channels, but they face challenges in processing morphology and quality. Hence, picosecond laser etching with galvanometer scanning is proposed to machine spiral microfluidic channels on a glass substrate. The objective is to disperse and sort microparticles from a glass microchip that is difficult to cut. First, the micropillar array and the spiral microchannel were designed to disperse and sort the particles in microchips, respectively; then, a scanning path with a scanning interval of 5 μm was designed according to the spot diameter in picosecond laser etching; next, the effects of laser power, scanning speed and accumulation times were experimentally investigated regarding the morphology of spiral microchannels; finally, the microfluidic flowing test with 5 μm and 10 μm microparticles was performed to analyze the dispersing and sorting performance. It was shown that reducing the laser power and accumulation times alongside increasing the scanning speed effectively reduced the channel depth and surface roughness. The channel surface roughness reached about 500 nm or less when the laser power was 9 W, the scanning speed was 1000 mm/s, and the cumulative number was 4. The etched micropillar array, with a width of 89 μm and an interval of 97 μm, was able to disperse the different microparticles into the spiral microchannel. Moreover, the spiral-structured channel, with an aspect ratio of 0.51, significantly influenced the velocity gradient distribution, particle focusing, and stratification. At flow rates of 300–600 μL/min, the microparticles produced stable focusing bands. Through the etched microchip, mixed 5 μm and 10 μm microparticles were sorted by stable laminar flow at flow rates of 400–500 μL/min. These findings contribute to the design and processing of high-performance glass microfluidic chips for dispersion and sorting.

## 1. Introduction

Spiral microfluidic channels exhibit significant potential for particle sorting, mixing and manipulation in biomedical engineering due to their unique hydrodynamic properties [1,2]. Through the centrifugal effect of fluids and flow gradients, spiral flow channels can efficiently focus, disperse and sort particles, providing an ideal platform for single-cell analysis, particle capture and biochemical reactions [3]. This sorting method has become a hot direction in microfluidic chip research because it significantly reduces energy consumption and system complexity without the need for complex driving devices such as external electric and magnetic fields [4].

The microfluidic chip is an innovative technology to accurately manipulate tiny liquid samples, which is widely used in medical diagnosis, drug screening and environmental monitoring [5,6]. The morphology and surface quality of microchannels are very important for separation efficiency [7]. However, the influence of chip materials, structural design and processing technology on the flow channel performance remains difficult and the focus of current research [8,9,10,11]. Glass material has become an important choice for microfluidic chips due to its excellent thermochemical stability and optical transparency, but its high hardness and fragility bring challenges to the processing of complex runner structures [12,13].

The performance of a spiral microfluidic channel depends not only on the design structural parameters but is also closely related to the processing technology. Traditional processing methods, such as ultrasonic processing [14], chemical etching [15] and abrasive jetting [16,17], are difficult to realize high-precision, complex-shaped runner processing on glass substrates. At present, ultra-short pulse laser technology has obvious advantages in the manufacture of glass microchannels because of its high precision and non-contact advantages [18,19,20]. Compared with femtosecond lasers, picosecond lasers have more practical application value in terms of cost and system stability [21]. Moreover, picosecond lasers can be obtained from semiconductor lasers using gain-switching technology, which is less costly and easier to have high pulse energy and high average power [22]. Jing et al. [23] used water-assisted picosecond laser processing to prepare microchannels with porous surfaces and almost no recast layers and cracks on zirconia ceramics. Compared with laser direct ablation, the depth increased by 52.74%, but the flow channel morphology was not neat. Fan et al. [21] used a picosecond laser with a pulse duration of 10 ps and wavelength of 355 nm to process microchannels, and they used the offset method and improved bidirectional method to plan the scanning path of the laser beam so as to obtain a symmetrical cross-section profile and smooth channel bottom. The microfluidic mixing channel with a width of 400 μm and a depth of 240 μm was successfully prepared, but the roughness at the bottom of the channel was 790~990 nm.

In this paper, we focus on the design and processing of a spiral flow channel with the demand of particle sorting in biomedicine as the starting point. A picosecond laser technology with a wavelength of 532 nm and a pulse width of 7 ps is used to optimize the processing morphology and surface quality of the spiral flow channel on the glass substrate by adjusting the process parameters such as the laser power, scanning speed, and the accumulation number under a galvanometer scanning system. The experimental results show that high-quality runner structures with complete runner structures and bottom roughness below 500 nm can be realized with appropriate processing parameters. In further flow performance tests, the focusing effect and sorting performance of the spiral flow channel under different flow rates and particle sizes are verified, revealing the significant effects of particle size and flow rate on the location of the focusing zone.

## 2. Microfluidic Structure Design and Laser Processing

### 2.1. Microfluidic Flow Channel Structure Design

**Figure 1:**
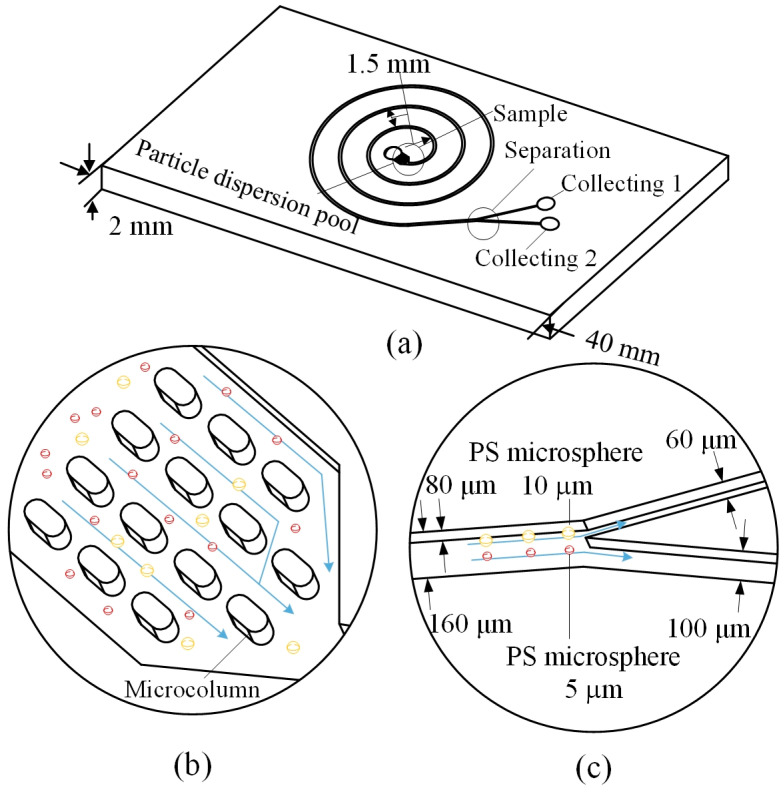
Micro–nano topology design diagram of microfluidic chip: (**a**) chip 3D model; (**b**) particle dispersion pool; (**c**) particle sorting port.

For the design of the cell-sorting microfluidic chip, its micro–nano topology is shown in Figure 1, which includes an inlet module, a spiral sorting module and a shunt module. Different cells are represented by microparticles of different colors and particle sizes. The inlet module is divided into five entrance channels by a microcolumn array. To effectively disperse particles and prevent excessive flow resistance, it is empirically determined that the dimensions of the micropillars should be 5~10 times the size of the particles with the channel spacing being at least three times the particle diameter. Based on this principle, the micropillars were designed with a length of 120 µm, a width of 60 µm and an interval of 90 µm. The initial radius of the spiral module is 1.5 mm, the interval of each circle is 1.5 mm, and there are three circles in total. The depth of the microfluidic channel *h* is 80 μm and the width of the channel *d* is 160 μm. At the sorting inlet, the channel is divided into two sub-fluidic channels with widths of 60 μm and 100 μm, respectively. The chip consists of a sample cell, a collection cell and other parts, which are connected by the flow channel. The working principle of the chip is as follows: firstly, the mixed cell solution is injected from the sample port, and the sample cells are guided into the spiral sorting module through the flow channel. In the spiral flow channel, due to the curvature-induced secondary flow (Dean flow) and inertia effect, cells of different sizes will migrate to different equilibrium positions in the cross-section of the flow channel, where the larger-sized cells tend to be close to the inner wall of the flow channel, and the smaller-sized cells tend to be on the outer wall. After passing through the spiral sorting module, cells of different sizes are guided to different sub-flow channels at the flow channel sorting port: large-sized cells enter the sub-flow channel with a width close to the inner wall, while small-sized cells enter the sub-flow channel close to the outer wall and flow into the corresponding collection cell for collection.

### 2.2. Microfluidic Chip Processing Materials and Experimental Conditions

The experimental material in this paper is 40 mm × 80 mm × 2 mm silica soda–lime glass, whose physical properties are shown in Table 1. Before the experiment, in a clean room environment, we used flatbed cleaning equipment to clean the glass surface dust and dirt to ensure that the glass upper and lower surfaces were clean so that the laser beam was well transmitted inside the soda–lime glass.

**Figure 2:**
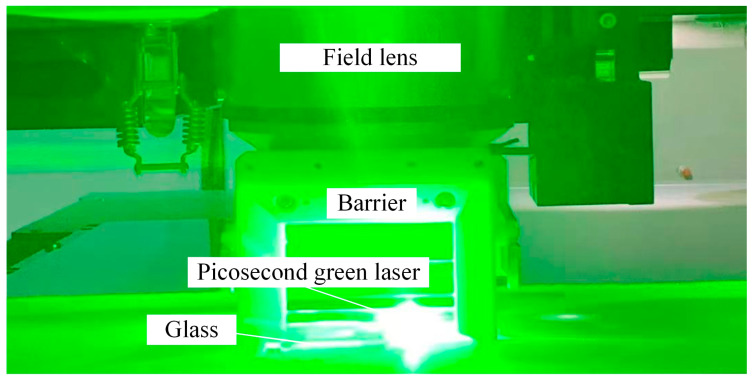
Picosecond green light etching of glass experimental scene.

**Figure 3:**
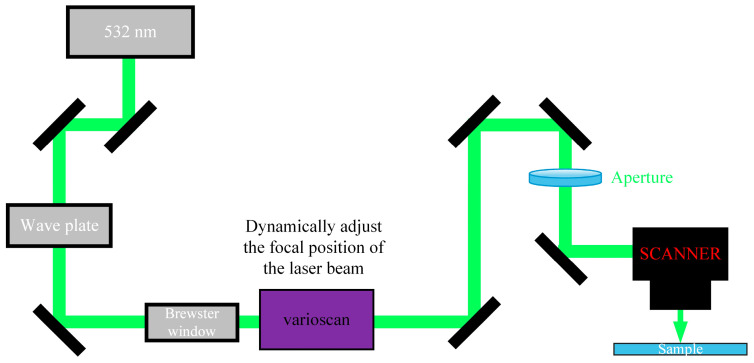
Schematic diagram of laser optical path.

Figure 2 shows the site diagram of the picosecond green light etching glass processing, and the experimental parameter conditions of the laser are shown in Table 2. Figure 3 shows a schematic diagram of the laser light path used by the laser to etch the glass microrunner, including the laser beam entering the scanning galvanometer (excelliSCAN scan heads) passing through the waveplates, the Brewster window, the varioscan (dynamic focusing system for laser processing and scanning) and aperture, and other components before it finally passes through the scanning galvanometer to form a stable laser spot acting on the surface of the glass. The laser (RX 532-40) has a center wavelength of 532 nm and a pulse duration of 7 ps. The scanning of the focused laser beam on the focal plane is controlled by the galvanometer. The laser beam scanning trajectory was programmed using scanning galvanometer software according to the graphic geometry and the moving strategy of the spot path.

### 2.3. Microfluidic Chip Laser Processing Method

**Figure 4:**
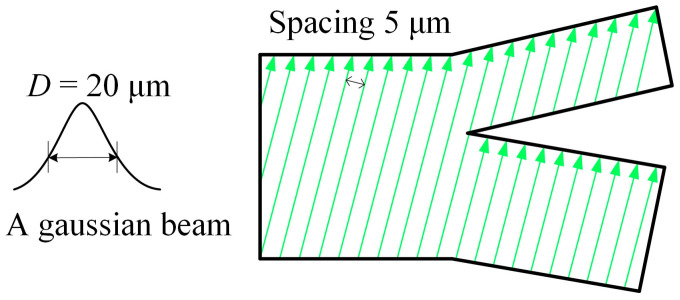
Processing path of laser etching channel.

Figure 4 shows the processing path diagram of the laser etching runner. Taking the machining path at the particle sorting port as an example, the spot diameter *D* of the laser is 20 μm. Because the spot energy presents Gaussian distribution, the parallel interval *S* between laser etching paths is designed to be 5 μm when machining microgrooves, so as to keep the energy distribution uniform. During processing, firstly, according to the CAD design drawing of the chip, the scanning path of the laser is filled and then the scanning galvanometer is introduced. The scanning galvanometer can control the laser to move in X and Y directions and realize the material removal. By increasing the scanning times *N*, the processing depth is increased to complete the processing of the chip.

To fabricate microchannels with the designed width specified in the schematic, path planning must account for practical machining conditions to correct for errors. The machining errors primarily arise from two factors. First, the movement of the laser spot is modeled as a single point, but the actual spot diameter is 20 μm. Consequently, during machining, the laser spot moving along the path exceeds the intended channel width, introducing errors. Second, variations in the absorption of laser energy at different wavelengths by the material result in inconsistent material removal, further affecting the channel width. To address these sources of machining error, it is common to compensate by scaling the dimensions of the design during path planning. The scaling factor is determined experimentally. Based on experimental results, using the picosecond green laser in this study to machine a channel with a target width of 100 μm on soda–lime glass reduced the design width to 75% of the original value and produced an actual channel width closest to 100 μm.

## 3. Numerical Simulation of Particle Motion in Microfluidic Chips

COMSOL Multiphysics 6.1, a multi-physics field simulation software based on the finite element method, is used to solve the velocity and pressure fields of incompressible Newtonian fluid flow and the trajectories of particles. Due to the low velocity, small size and low Reynolds number of the microchannel, the laminar flow module is used to solve the Navier–Stokes (N-S) equations and continuity equations to obtain the fluid velocity and pressure fields. The N-S and continuity equations for the control fluid are as follows.

(1) $$\begin{array}{c} \nabla (u)=0\\ \rho (u\nabla )u=\nabla [pI+\mu (\nabla u+{\left(\nabla u\right)}^{T})]+F \end{array}$$

where u is the velocity field, ρ is the fluid density, μ is the dynamic viscosity, and F is the volume force. The laminar flow module uses P2 + P2 (second-order velocity and second-order pressure) to discretize the fluid, the fluid density and the kinetic viscosity are the corresponding properties of water in the built-in material, the wall boundary condition is no slip, the inlet boundary condition is fully developed flow, and the outlet boundary condition is 0 hydrostatic pressure to inhibit backflow. After obtaining the velocity field of the fluid flow by steady-state solving, the particle trajectories are computed using the fluid flow particle tracking module based on the following equations of motion.

(2) $$\frac{d}{dt}(m_{p}v)=F_{D}+F_{G}+F_{ext}$$

where *m_p_* denotes is the mass of the particle, **v** is the particle velocity, **F***_D_* is the drag force, **F***_G_* is the gravity force, and **F***_ext_* is other external forces on the particle. The fluid flow particle tracking module uses a bouncing wall condition where the trailing force is imposed by Stokes’ law incorporating the wall modification due to the low Reynolds number in the channel. Since the Saffman lift can be considered zero for buoyant particles in a Poisson lobe flow, the wall-induced lift law is used to apply lift to the particles. The flow rate starts at 400 μL/min. Fifty particles of sizes 5 and 10 were released simultaneously from the inlet at particle time 0. The particles were released from the inlet at the same time as the particles were released from the inlet.

## 4. Microfluidic Chip Packaging and Flow Performance Testing

### 4.1. Packaging of Microfluidic Chips

**Figure 5:**
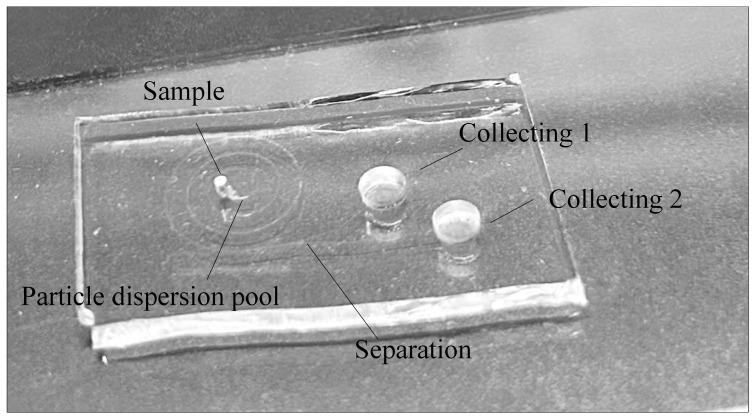
Physical encapsulated glass microfluidic chip.

Figure 5 shows the actual glass microfluidic chip after the encapsulation is completed. The glass microfluidic chip is encapsulated by the method of plasma bonding. PDMS material was selected as the encapsulation material, and plasma treatment was performed before encapsulation. Since punching was performed after the encapsulation was completed, the PDMS material debris entered the flow channel and would cause blockage. Therefore, before the plasma treatment, it is necessary to use a carbon dioxide laser to punch the inlet and outlet holes of the PDMS material. Then, after cleaning the perforated PDMS material, the PDMS material and the glass chip were divided into laminating and non-laminating surfaces, and plasma treatment was performed on the laminating surface for 30 s. Finally, the laminating surfaces of the glass chip and the PDMS material were bonded and kept warm for 4 h at 130 degrees Celsius by using a thermostat.

### 4.2. Microfluidic Chip Detection Platform and Detection Method

**Figure 6:**
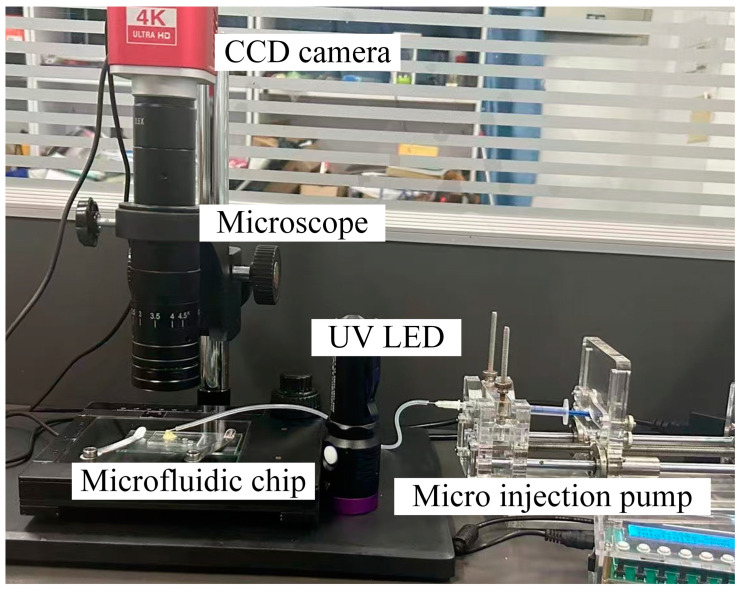
Chip flow performance testing platform.

Figure 6 shows the self-constructed chip testing platform, which mainly includes a CCD camera (luosi company), microscope, carrier stage, micro-syringe pump (yuanhang company) and excitation light source (fenghuo company). Fluorescent polystyrene microparticles of different colors and particle sizes (later called fluorescent microparticles), instead of cells of different sizes, were used to test the sorting function of the spiral flow channel chip. In the spiral flow channel, fluorescent microparticles with different particle sizes migrate to different locations in the flow channel due to the inertia effect and secondary flow effect of the fluid. At the exit of the flow channel, under the excitation light source, different fluorescent particles in the chip show different colors, and the distribution of fluorescent particles in the sorting port is observed and recorded by microscope. The positional distribution of the fluorescent particles in the flow channel was photographed using an industrial-grade microcamera combined with a fluorescence microscope to evaluate the sorting performance of the spiral flow channel on the fluorescent particles.

## 5. Results and Discussion

### 5.1. Determination of Laser Process Parameters and Experimental Analysis

When a picosecond green light was used to etch microfluidic channels on glass, the laser process parameters were further optimized in order to find the optimal parameter combinations for the runner processing quality under the combined influence of the laser power *P*, scanning speed *v_l_*, and the number of scans *N*. The laser process parameters were then optimized by using the laser power as the test factor. The above three laser processing parameters were used as test factors, four level values were set under each test factor, and finally a three-factor, four-level orthogonal test was designed. The orthogonal table was chosen as L16(4^5^), and the process parameter levels were selected as shown in Table 3.

**Figure 7:**
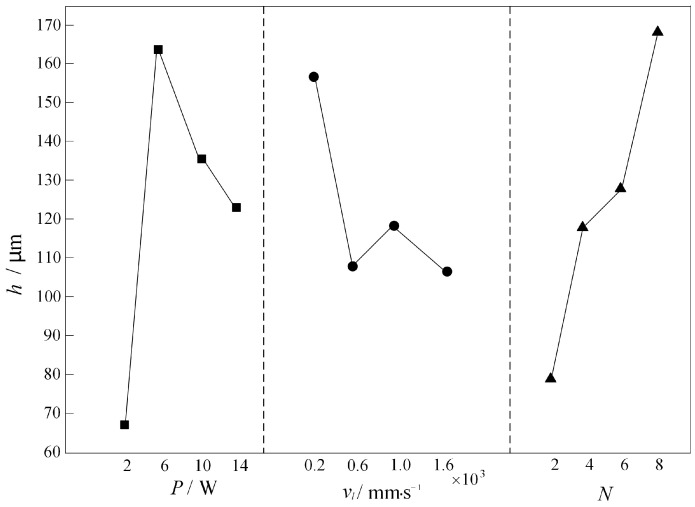
Relationship between *P*, *v_l_*, and *N* at different levels and channel depth *h*.

Figure 7 shows the laser power *P*, scanning speed *v_l_* and number of scans *N* versus the processing depth *h* at each level in the orthogonal test table. It can be seen that the mean value of the microfluidic channel depth *h* increases with the increase in the accumulated number of times *N* and decreases with the increase in the scanning speed *v_l_*. As the laser power *P* increases, the runner depth increases and then decreases, but when the power is too high, the depth decreases and there is melt; this is because the high-power laser input energy exceeds the evaporation or ablation threshold of the glass, resulting in a rapid thickening of the molten layer on the surface of the glass. Due to the poor fluidity of the molten glass, the liquid glass formed at high temperatures cannot be quickly discharged from the flow channel, leaving a large amount of molten material in the flow channel. Among the three laser processing parameters, the one that has a greater influence on the runner depth *h* is the number of scans *N*. The runner depth is maximized when *N* = 8. In addition, the process parameter combinations that maximized the microfluidic channel depth were *N* = 8, *v_l_* = 200 mm/s, and *P* = 6 W.

**Figure 8:**
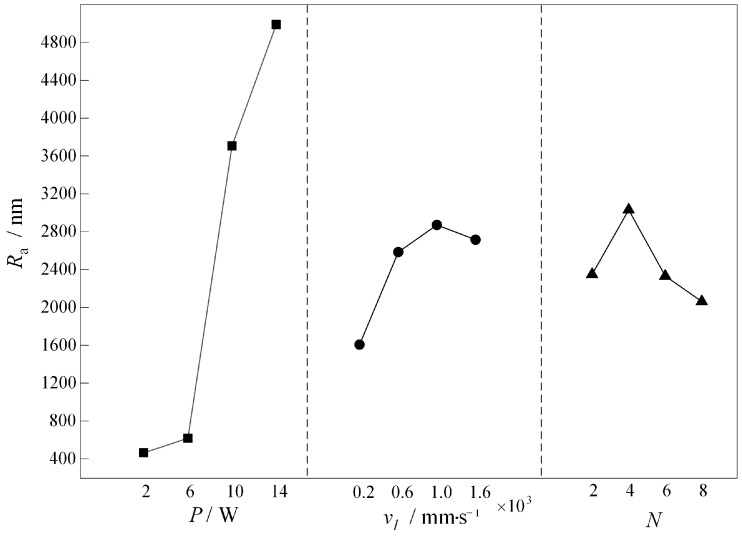
Relationship between *P*, *v_l_*, and *N* at different levels and surface roughness *R*_a_.

Figure 8 shows the relationship between the laser power *P*, scanning speed *v_l_*, scanning times *N* and the surface roughness *R*_a_ of the runner at each level in the orthogonal test table. It can be seen that the surface roughness *R*_a_ increases with the increase in laser power *P*, but it has a decreasing trend with the increase in laser scanning speed *v_l_* and the number of scanning times *N*. The surface roughness *R*_a_ of the runner surface increases with the increase in laser scanning speed *v_l_* and the number of scanning times *N*. Among the three laser processing parameters, the one that has a greater influence on the surface roughness *R*_a_ is the laser power *P*. In addition, the combination of process parameters that minimizes the surface roughness *R*_a_ is *N* = 8, *v_l_* = 200 mm/s, and *P* = 2 W.

**Figure 9:**
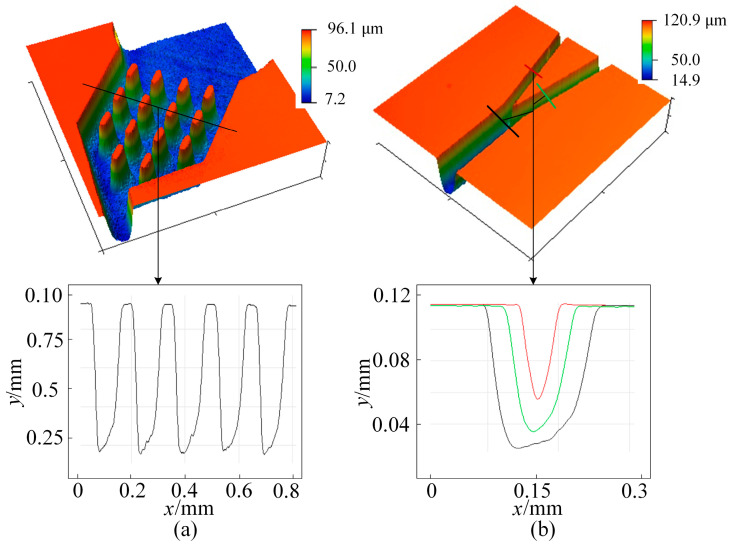
Three-dimensional (3D) morphology detection of microfluidic chips: (**a**) structural morphology of micropillar array; (**b**) sorting port channel morphology and contour.

Our analysis of runner depth and runner roughness showed significant results. At the repetition frequency *f* of 200 kHz, the selection interval of laser power *P* is set as 6~10 W, the scanning speed *v_l_* is controlled at 600~1000 mm/s, and the number of scans *N* is 4~6. Therefore, when machining a runner with a runner width *d* = 160 μm on glass, the excitation frequency f = 200 kHz is selected, and the laser power *P* = 9 W, *v_l_* = 1000 mm/s and *N* = 4 are taken in the above range. The surface topography of the processed microfluidic chip is examined by laser confocal microscopy, and its 3D topography is shown in Figure 9. The depth and roughness can be measured by using the two functions’ Extract profiles along arbitrary lines, and we calculated the roughness parameters in the tools directory of Gwyddion (2.6.0) software. Figure 9a shows the three-dimensional profile inspection map of the micropillar array processed for microparticle dispersion and prevention of clogging, and it can be seen that the columns are 50 μm wide, 97 μm apart, and 89 μm deep with a complete structure and relatively uniform bottom depth. Figure 9b shows the microparticle sorting port, channel depth 163 μm, width 83 μm, and depth–width ratio 0.51. The surface roughness *R*_a_, the bottom of which is in the range of 340~500 nm, improves the roughness by about 40% compared with that of UV picosecond laser-etched glass runners, and it can be further enhanced by polishing [24,25]. The cross-sectional shape of the runner is V-shaped, and this structure is more important for subsequent observation and accuracy of sorting and analysis.

### 5.2. Simulation Results of Particle Motion on Microfluidic Chips

**Figure 10:**
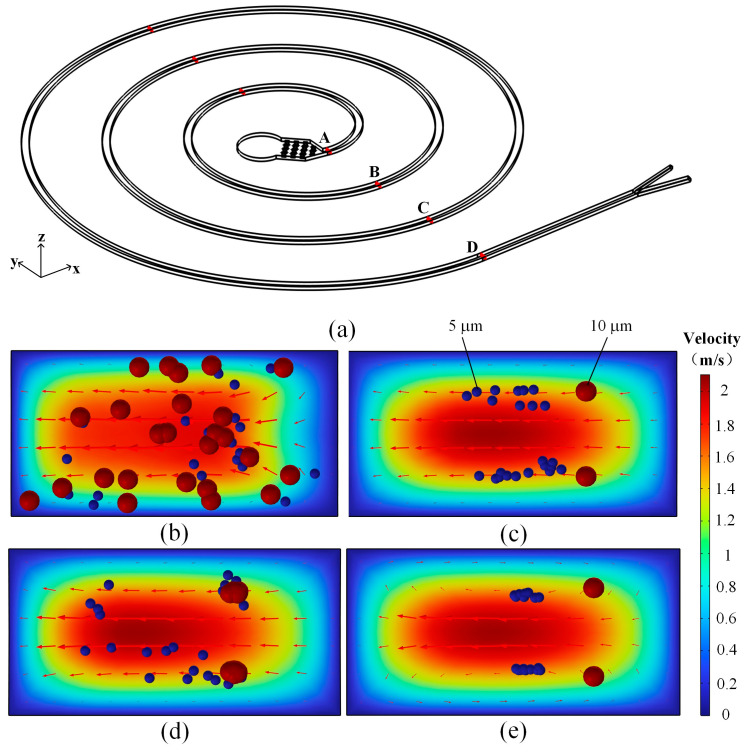
Cross-sectional view of microfluidic channel simulation: (**a**) 3D spiral microfluidic channel; (**b**) section A; (**c**) section B; (**d**) section C; (**e**) section D.

Figure 10 shows the state of large and small particles flowing through sections A, B, C and D when the inlet velocity is 400 μL/min. At the entrance, cross-section A is released by a mixture of particles of different sizes, which occupies a chaotic and disordered position, and under the influence of centrifugal force, the velocity field points to the positive direction of the *y*-axis. With the accumulation of turns, at section B, the large particles are primarily focused on the inside of the flow channel, and the small particles are primarily focused away from the inside of the flow channel. Affected by the flow field, the small particles move forward to the *y*-axis, and the small particles in section C are distributed in disorder, while the large particles are stable at the inner wall of the flow channel. When moving to section D, the velocity field presents a vortex with up-and-down reverse symmetry, which is the Dean vortex. Particles in the lift and Dean force gradually balanced under the action of focusing, large particles are affected by the Dean force relative to small particles, the double line focusing on the long side of the inner wall near the small particles focusing on the band is relatively wider, and the movement is away from the inner wall of the position. The large particles focus near the inner wall, and the small particles move away from the inner wall.

**Figure 11:**
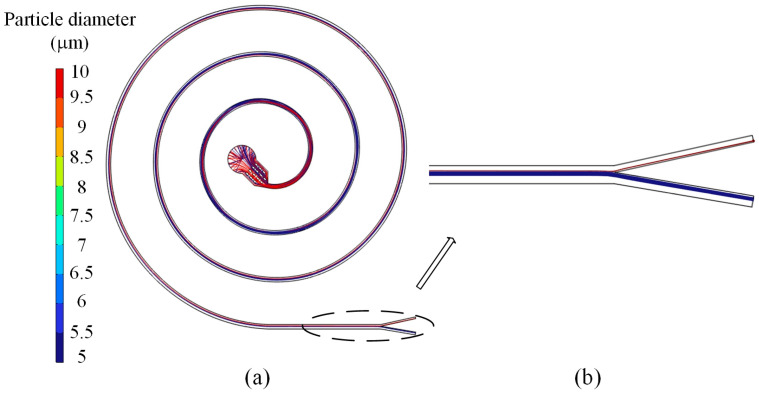
Simulation results of particle motion in x-y plane: (**a**) particulate motion result; (**b**) partial enlargement.

As shown in Figure 11, for the x-y plane particle motion simulation results, particles from the initial chaos transition to large particles near the inner wall, and small particles move away from the inner wall and the particle layered state. Ultimately, from the local magnification of the map, it can be observed that the particles are separated into different outlets.

### 5.3. Microfluidic Chip Performance Testing

**Figure 12:**
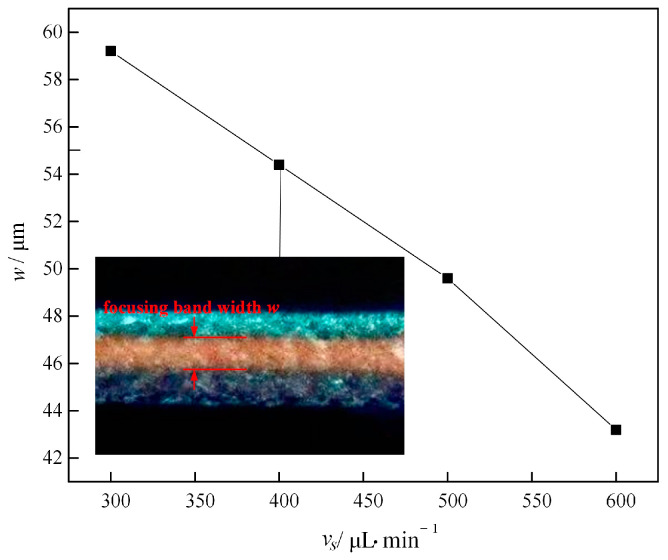
The relationship between the focusing bandwidth *w* of microparticles and flow rate (5 μm fluorescent particles).

Figure 12 shows a plot of the focusing band width *w* versus the inlet flow rate for 5 μm fluorescent microparticles in the flow channel. With the gradual increase in the velocity from 300 μL/min to 600 μL/min, the width of the focusing band decreases from 59.2 µm to 43.2 µm, which indicates that the increase in the injection velocity effectively compresses the width of the focusing band of the microparticles. With the increase in flow velocity, the inertial lift and Dean drag also increase, and the focusing intensity further increases, so the focusing bandwidth is compressed.

**Figure 13:**
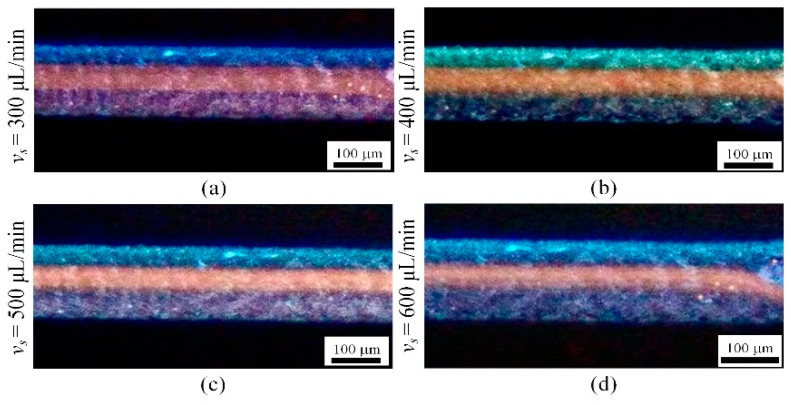
Focusing results for 5um particles(red): (**a**) flow rate of 300 μL/min; (**b**) flow rate of 400 μL/min; (**c**) flow rate of 500 μL/min; (**d**) flow rate of 600 μL/min.

Figure 13 presents the focusing position of 5 μm fluorescent particles. At a flow rate of 300 μL/min, the particle motion is primarily governed by Dean drag forces. Under the influence of Dean vortices, 5 μm particles focus at positions away from the channel walls. As the flow rate increases, the particle focusing position shifts with the vortices, gradually migrating toward the center of the channel.

**Figure 14:**
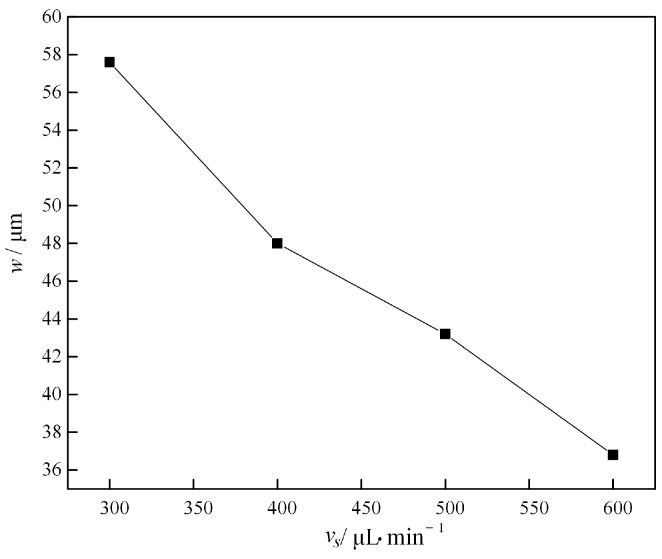
The relationship between the focusing bandwidth *w* of microparticles and flow rate (10 μm fluorescent particles).

**Figure 15:**
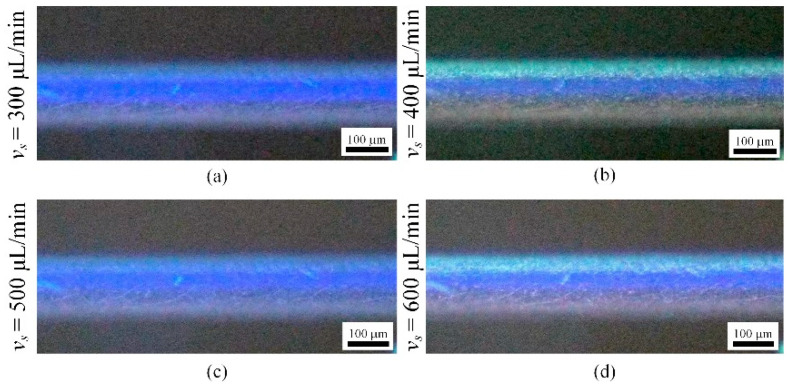
Focusing results for10 μm fluorescent particles(blue): (**a**) flow rate of 300 μL/min; (**b**) flow rate of 400 μL/min; (**c**) flow rate of 500 μL/min; (**d**) flow rate of 600 μL/min.

Figure 14 shows a plot of the focusing band width *w* versus flow rate for 10 µm fluorescent microparticles in the flow channel. As the flow rate increases from 300 μL/min to 600 μL/min, the width of the focusing band decreases from 57.6 µm to 36.8 µm. The width of the focusing band of the 10 µm microparticles is narrower and varies more significantly with the flow rate than that of the 5 µm microparticles. Figure 15 shows the focusing results of 10 μm fluorescent microparticles. The focusing position is located near the inner wall, which is due to the fact that the migration of larger particles is mainly controlled by inertial lift, and the influence of Dean drag is relatively small.

**Figure 16:**
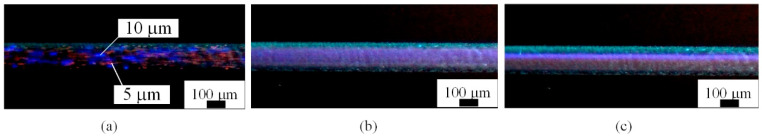
5 μm (red) and 10 μm (blue) particles stratification process: (**a**) 2 s before stabilization; (**b**) 1 s before stabilization; (**c**) stabilization.

After 5 μm and 10 μm microparticle focusing experiments, the two kinds of particles were fully mixed and injected into the flow channel at a step-by-step increasing flow rate. Figure 16 shows the process of gradual stratification of mixed microparticles. Figure 16a shows that when the flow rate is stabilized at the first 2 s of 1000 μL/min, the microparticles are dispersed through the micropillar array, while 5 μm microparticles (red) and 10 μm microparticles (blue) are mixed in disorder and flow to the outlet under the inlet pressure. Figure 16b shows that at the first 1 s of stabilization, the flow velocity increased, the clear microparticles could not be captured, and the mixing streamline was visible without stratification. Figure 16c shows that when the flow velocity is stable, obvious stratification can be observed: 10 μm particles are stable inside the flow channel, and 5 μm particles are stable at a position far away from the inside of the flow channel.

**Figure 17:**
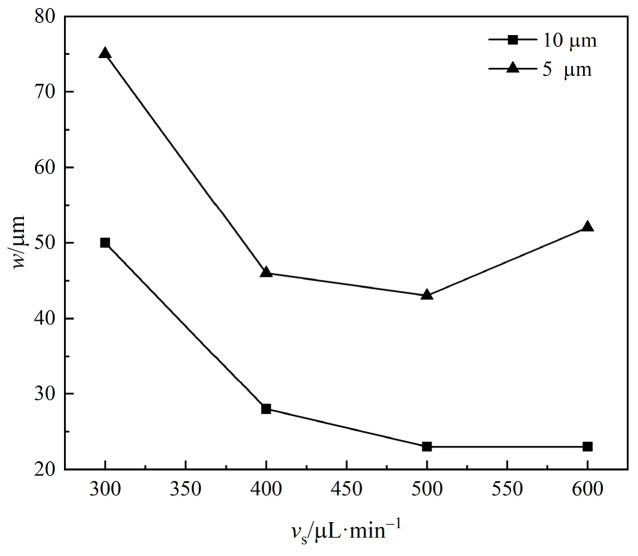
The relationship between the focusing bandwidth *w* of microparticles and injection flow rate.

In order to obtain the best stratification velocity, the velocity was increased step by step to observe the width of the microparticle focusing zone and the stratification phenomenon. As shown in Figure 17, with the increase in flow rate, the focus band width of 5 μm microparticles decreased significantly to 46 μm at 400 μL/min, slightly decreased at 500 μL/min and then began to increase. Therefore, for 5 μm microparticles, the optimal focusing flow rate is 500 μL/min. The change trend of the focus band of 10 mm microparticles is similar to that of 5 μm microparticles, and it drops to 28 μm at 400 μL/min, and then drops slightly at 500 μL/min, and then does not change. Therefore, 500 μL/min is the best focusing velocity for 10 μm microparticles.

**Figure 18:**
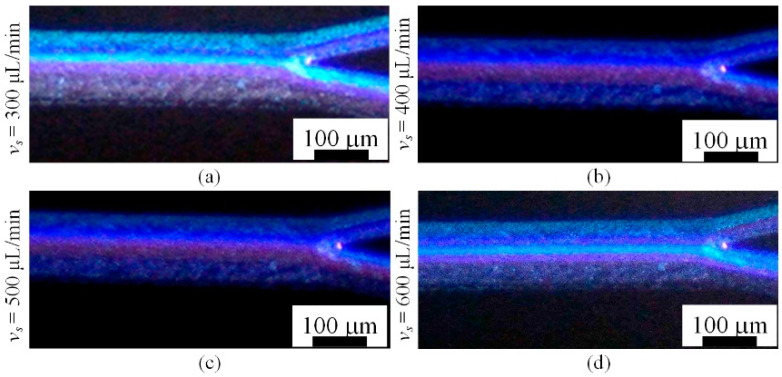
Results of stratification of 5 μm (red) and 10 μm (blue) mixed particles (**a**) flow rate of 300 μL/min; (**b**) flow rate of 400 μL/min; (**c**) flow rate of 500 μL/min; (**d**) flow rate of 600 μL/min.

From the stratification point of view, with the increase in flow velocity, as shown in Figure 18, when 400 μL/min and 500 μL/min, the stratification is obvious and the boundary is clear. According to the camera observation frame rate, the sorting time is about 2.88 s at a 400 μL/min flow rate. After the flow velocity rises again, the 10 mm and 5 mm particles begin to focus on the center of the flow channel, and the focusing positions coincide, so there is no stratification phenomenon: that is, they cannot be sorted.

## 6. Conclusions

(1) A 7 ps green laser was used to directly etch the micro–nano runners, and the results showed that the laser power was the most important factor affecting the surface roughness and depth of the glass microrunners by selecting a lower power in order to obtain a high quality of the processed surface. Meanwhile, the runner depth was increased by decreasing the scanning speed and increasing the number of scans. When the scanning path interval is set to 5 μm, the laser power used is 9 W, the scanning speed is 1000 mm/s, and the cumulative number of scans is four times, a high-quality micro–nano runner without edge defects can be obtained, and the bottom roughness is less than 500 nm.
(2) The experimental inspection and simulation of the flow channel show that the particle size and flow rate have a significant effect on the width and position of the focusing band of microparticles. The 5 μm microparticles are influenced by the Dean force, and the focusing position is far from the inner wall of the flow channel. As the flow rate increases, the aggregation position migrates with the Dean vortex and gradually moves to the center of the flow channel
(3) The 10 μm microparticle is dominated by inertial lift, its focus band is located near the inner wall of the flow channel, and its width also decreases with the increase in flow velocity. The mixed microparticles have stable and clear delamination at 400 μL/min and 500 μL/min. This characteristic provides an experimental basis for the sorting of microparticles based on particle size. This also shows that the separation of 5 μm and 10 μm microparticles can be achieved by adjusting the flow rate.

## Figures and Tables

**Table 1:** Material characteristics of silica sodium calcium glass.

Density/ (g/cm^3^)	Elastic Modulus/GPa	Linear Expansion Coefficient/°C^−1^	Vickers Hardness HV/GPa	Transition Temperature T_g_/°C	Poisson’s Coefficient	Toughness K_IC_/MPa·m^0.5^
2.5	72	9 × 10^−6^	2.5	550	0.22	0.75

**Table 2:** Experimental equipment process parameter conditions.

Wavelength/nm	Laser Power *P*/W	Spot Diameter *D*/μm	Repetition Frequency *f*/kHz	Scanning Speed *v_l_*/(mm·s^−1^)	Pulse Width/ps
532	0~18	20	100~500	0~4500	7

**Table 3:** Laser etching channel process parameter.

Parameter	Level 1	Level 2	Level 3	Level 4
Laser power *P*/W	2	6	10	14
Scanning speed *v_l_*/(mm·s^−1^)	200	600	1000	1600
Number of scans *N*	2	4	6	8

## Data Availability

The original contributions presented in this study are included in the article. Further inquiries can be directed to the corresponding author(s).
